# Adsorption and Self-Assembly of Large Polycyclic Molecules on the Surfaces of TiO_2_ Single Crystals

**DOI:** 10.3390/ijms14022946

**Published:** 2013-01-30

**Authors:** Szymon Godlewski, Marek Szymonski

**Affiliations:** Faculty of Physics, Astronomy and Applied Computer Science, Jagiellonian University, Reymonta 4, 30-059 Krakow, Poland; E-Mail: ufszymon@cyf-kr.edu.pl

**Keywords:** organic molecules, titanium dioxide, self-assembly, polycyclic molecules, adsorption

## Abstract

Titanium dioxide is one of the most frequently studied metal oxides, and its (110) rutile surface serves as a prototypical model for the surface science of such materials. Recent studies have also shown that the (011) surface is relatively easy for preparation in ultra-high vacuum (UHV) and that both the (110) and (011) surfaces could be precisely characterized using scanning tunneling microscopy (STM). The supramolecular self-assembly of organic molecules on the surfaces of titanium dioxide plays an important role in nanofabrication, and it can control the formation and properties of nanostructures, leading to wide range of applications covering the fields of catalysis, coatings and fabrication of sensors and extends to the optoelectronic industry and medical usage. Although the majority of experiments and theoretical calculations are focused on the adsorption of relatively small organic species, in recent years, there has been increasing interest in the properties of larger molecules that have several aromatic rings in which functional units could also be observed. The purpose of this review is to summarize the achievements in the study of single polycyclic molecules and thin layers adsorbed onto the surfaces of single crystalline titanium dioxide over the past decade.

## 1. Introduction

In recent years, there has been increasing interest in the adsorption of organic molecules on crystalline substrates. This interest is driven by both the fundamental aspiration to investigate the elementary processes involved in the adsorption, diffusion and self-assembly of these molecules and by the considerable diversity of their practical applications. Therefore, many new experimental and theoretical studies focusing on organic molecules on surfaces are reported every year. A considerable amount of effort is focused on investigating the electrical, optical and chemical properties of these molecules and on the binding mechanisms. Similarly, the nanostructuration of ordered frameworks induced by both self-assembly processes and lateral manipulation by scanning probe microscopy also attracts considerable attention. In this context, self-assembly provides a powerful and unique method for fabricating well-ordered nanostructures on large sample areas in a parallel one-step technique in which there is no need to perform sequential actions. Indeed, self-assembly provides a relatively easy and cheap technique for nanostructuring, especially compared to sequential single molecule manipulation actions. Therefore, there have been many studies on determining the effective self-assembly growth parameters, e.g., the substrate temperature during adsorption or post-deposition thermal annealing. To date, the majority of experiments have been performed on metal substrates. However, due to their unique properties, the incorporation of semiconductors and insulators as templates for molecular growth has recently been observed, although the common presence of surface defects or dangling bonds exposed at the surface of the substrate makes the processes even more complicated.

Among the various available substrates, metal oxides play a remarkable role, and titanium dioxide is often considered a model surface. The popularity of titanium dioxide arises from its wide range of practical applications, which range from catalysis, coatings, and the fabrication of sensors to usage in the optoelectronic and medical industries [1–12]. Due to its scientific and practical importance, the self-assembly of organic molecules is one of the most rapidly developing research fields in the surface science of titania.

Among the three structural phases of titanium dioxide (*i.e.*, rutile, anatase, and brookite), rutile is the most studied in the context of organic molecule adsorption, which is due to its relatively ready availability. However, when titanium dioxide nanoparticles are created, the anatase form is adapted, which indicates that it is the lowest energy structure [13–15]. Because of the difficulties in obtaining high quality single-crystalline anatase, almost all experiments with organic molecules are conducted on different rutile faces. However, in recent years, there has been increasing interest in investigating the behavior of molecules on anatase crystals, and several experiments with organic species have been performed [16–18]. In contrast, large organic polycyclic molecules have only been investigated on rutile surfaces to date. Calculations indicate that ideal rutile crystals are terminated by the (110), (100) and (011) faces [19]. In many applications where the titanium dioxide is prepared in a powder form by the oxidation of titanium tetrachloride, both the (110) and (011) faces are present, which has been confirmed by scanning and transmission electron microscopy measurements [20]. The majority of the research on organic molecules has been performed on the (110) surface, which serves as a model system for comprehending the physics and chemistry of transition metal oxide surfaces; however, there are examples of experiments performed on (011), cross-linked (110) and other surfaces.

Molecular layers, multilayers and nanostructures formed on titanium dioxide surfaces modify the surface properties that leads to numerous applications in photovoltaics, optoelectronics, gas sensing, catalysis and in medicine. Therefore nowadays the structural, chemical and electronic properties of molecular assemblies on titanium dioxide surfaces attract growing attention of scientist all over the world.

In this review, we will focus on the adsorption and self-assembly of relatively large organic polycyclic molecules on vacuum-prepared single-crystalline TiO_2_ surfaces and the developments achieved over the last decade. We will omit all smaller molecular species, which have also been thoroughly investigated and whose detailed descriptions can be found in comprehensive reviews by Diebold [1], Pang [21] and the recent review by Thomas and Syres [13]. Before describing the details on the adsorption and self-assembly of large polycyclic molecules, we shall begin with a brief introduction of the most frequently studied TiO_2_ surfaces, their geometrical structures and electronic properties.

## 2. TiO_2_ Surfaces as Templates for the Adsorption of Organic Molecules

The well-established methodology of preparing TiO_2_ single crystal surfaces is based on ultra-high vacuum cyclic sputtering with Ar^+^ ions and annealing at temperatures of approximately 700 °C. In these processes, the crystals are reduced by introducing oxygen vacancies with new states located in the intrinsic band gap. The concentration of vacancies can be tuned by appropriately controlling the annealing temperature and the duration of sputtering time. During the reduction process, the color of the titanium dioxide samples changes from a semi-transparent, light cream color into yellow, blue, dark navy-blue to almost black, where the concentration of vacancies is the greatest. In this review, we will focus on three different surfaces of rutile titanium dioxide, including the (110), (011) and cross-linked (110) surfaces, because almost all experiments with polycyclic molecules have been conducted on these surfaces.

**Figure 1:**
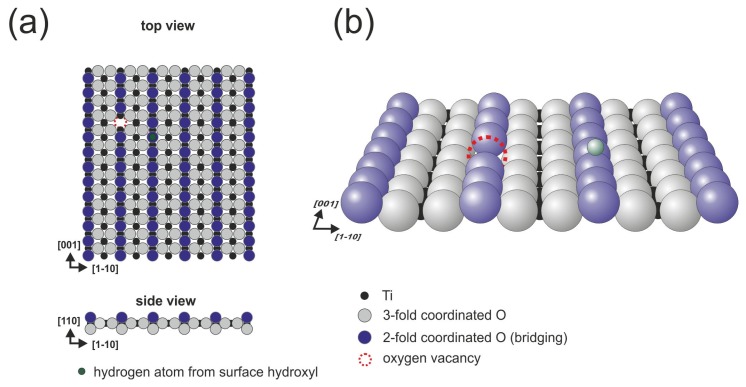
Rutile TiO_2_(110)-(1 × 1) surface: (**a**) top view, and (**b**) perspective view.

The (110) surface contains characteristic bridging oxygen rows (violet balls in Figure 1), in-plane oxygen ions (gray balls in Figure 1) and five-fold coordinated titanium atoms (Ti_5c_, black balls in Figure 1), which play a leading role in the binding of several organic species (see [13,22] and references therein). It is well-known that the history of the sample has a meaningful influence on its structure because the concentration of oxygen vacancies increases during consecutive preparation cycles. The vacancies are formed not only in the bulk but also on the surface within the bridging oxygen rows (red dashed circle in Figure 1), both introduce new states at approximately 1 eV below the Fermi level [13,23]. When exposed to residual water, the oxygen vacancies are filled with dissociated water molecules that form the so-called surface hydroxyls (the hydrogen atom from the surface hydroxyl is marked in green in Figure 1). The structure of the (110) surface has been confirmed by numerous spectroscopic and scanning probe microscopy techniques. During STM (scanning tunneling microscopy) imaging, only empty states could be stably recorded, and the bright contrast that corresponds to the elevated altitude of the STM tip is recorded over 5-fold coordinated titanium atom rows that are situated in the surface trenches, contrary to the geometrical structure in which the bridging oxygen atoms are exposed from the surface plane. This fact strongly indicates that electronic effects play an important role during the STM imaging of TiO_2_ surfaces.

**Figure 2:**
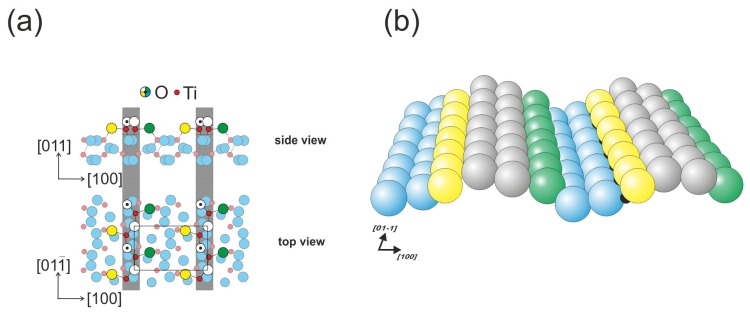
Rutile TiO_2_(011)-(2 × 1) surface: (**a**) top view and (**b**) perspective view.

The (011) face of rutile titanium dioxide is the third most stable [19], and it undergoes reconstruction into the (2 × 1) phase during preparation. The structural model of the surface has been the subject of vibrant debate over the years. The latest model introduced in 2008 by Torrelles *et al.* and Gong *et al.* has been confirmed by SPM (scanning probe microscopy), LEED (low energy electron diffraction) and surface X-ray diffraction methods [24,25]. According to this model, the surface is composed of double outermost oxygen rows that form zig-zag patterns (Figure 2). The five-fold coordinated titanium atoms are slightly hidden, which may block their role in the formation of chemical bonds with adsorbates [26]. The STM images of the (011) surface strongly depend on the tunneling junction settings, and the surface is recorded as a zig-zag pattern on top of the outermost rows for small biases. For larger biases, the pattern evolves into ellipse-like features. The surface appears to be resistant to the formation of surface oxygen vacancies [13]; however, hydroxyl groups formed by hydrogen atoms bound to oxygen atoms are also frequently observed on the surface.

**Figure 3:**
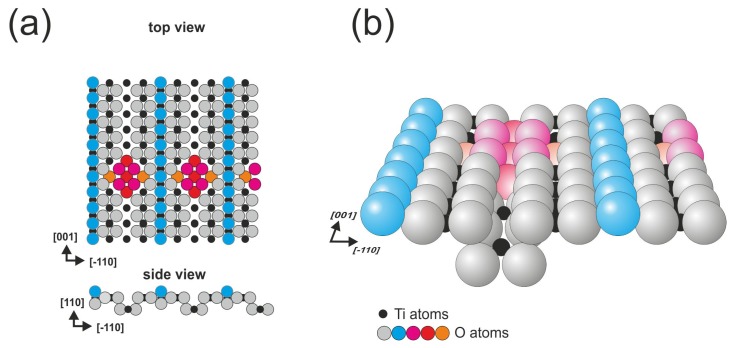
Rutile TiO_2_(110)-(2 × 1) surface: (**a**) top view and (**b**) perspective view; oxygen atoms forming cross-links are marked in orange, red and purple.

The third surface described in this work is obtained from the same crystal as the previously described (110) face, but the cross-linked surface is formed when the reduction level is increased; the cross-linked structure is not present alone, it coexists with the (1 × 1) and (2 × 1) reconstructions. The two most often described models of the surface are based on Ti_3_O_6_ and Ti_2_O_3_ motifs. nc-AFM (non-contact atomic force microscopy) measurements performed by Pieper *et al.* strongly supported the Ti_3_O_6_ model and revealed that the Ti_2_O_3_ structure is inconsistent with their measurements [27]. In Figure 3, we present the Ti_3_O_6_ based model that is consistent with SPM experiments.

In addition to the three surfaces, other faces of rutile titanium dioxide, such as (100) and (001), and anatase crystals have also been used for the deposition of organic species; however, only inorganic and smaller non-polycyclic molecules have been investigated to date.

We will begin our discussion of experimental studies with polycyclic molecules using pure hydrocarbon molecules that were synthesized in the form of polyaromatic platforms.

## 3. Planar Polyaromatic Hydrocarbons

**Figure 4:**
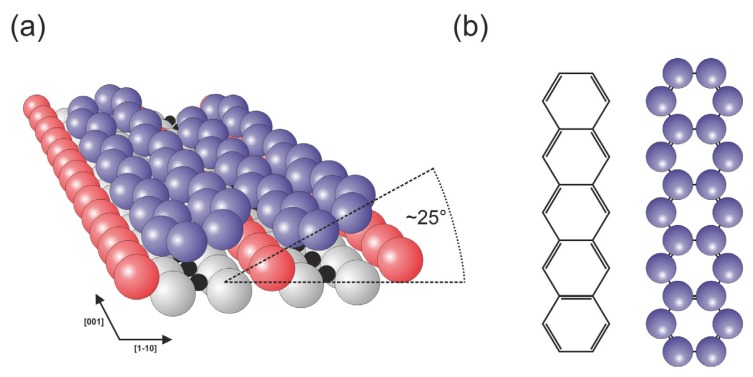
Pentacene layer on the TiO_2_(110) surface; the molecules lie flat in a geometry that is tilted by approximately 25° with respect to the surface plane to form molecular lines that run essentially perpendicular to the rows of bridging oxygen atoms. (**a**) perspective view [35]; (**b**) pentacene molecule; color coding: red—bridging oxygen, light gray—remaining surface oxygen atoms, black—titanium, violet—carbon, for clarity hydrogen atoms in the pentacene molecule are not shown.

Among the various planar hydrocarbons, pentacene molecules are treated as archetypical polyaromatic species, and their behaviors have been investigated on various substrates that exhibit metallic, semiconducting and insulating characteristics [28–30]. Pentacene molecules have been used to construct organic field-effect transistors with high carrier mobility [31] and high efficiency organic photovoltaic devices [32–34]. Lanzilotto *et al.* have investigated the self-assembly of pentacene molecules on the TiO_2_(110) surface using NEXAFS (near-edge X-ray absorption fine structure) spectroscopy and STM measurements [35]. The molecules were observed to be physisorbed on the surface with their longer axis parallel to the rows of surface oxygen atoms. With increasing coverage, the molecules tend to form molecular stripes that run essentially perpendicular to the rows of surface oxygen atoms, *i.e.*, in the [1–10] direction in a similar manner to that recently reported for the PTCDA case [36]. The presence of the surface bridging oxygen rows influences the periodicity of the molecules within the stripes, which lead together with the side-by-side pentacene attraction to the commensurate spacing along the [1–10] direction, where the neighboring molecules are separated by a distance that is equivalent to the separation of the rows of surface oxygen atoms. Within the monolayer, the molecules lie on the surface in a geometry that is tilted by approximately 25° around the molecular axis, as illustrated in Figure 4.

In contrast, the spacing of molecular lines along the rows of bridging oxygen atoms is non-uniform and driven by molecule-molecule repulsion. Therefore, the structure exhibits no commensurability along the surface rows; furthermore, with increasing coverage, the spacing of molecular lines decreases and they become less meandering as they approach the [1–10] direction. In principle, the weak binding with the substrate conjugated with relatively strong intermolecular interactions and tilting of neighboring molecules, which makes the structure of the film similar to the pentacene layers in the bulk, results in the development of bulk-like electronic properties in a layer. The flat-lying manner of adsorption is observed up to the 3rd layer, but the presence of molecular lines is not preserved [35].

Potapenko *et al.* [37] have investigated the adsorption of anthracene on the (110) surface using STM and TPD (thermal programmed desorption) techniques. The TPD spectra indicated that the molecules desorb from the multilayers at 270 K and from the first layer at approximately 360 K, therefore only a monolayer could be formed at room temperature. The molecules were observed to adsorb in a flat-lying geometry with their longer axis parallel to the rows of bridging oxygen atoms in the trenches between the rows. The incommensurate structure of the monolayer was formed due to vdW (van der Waals) repulsion between neighboring molecules adsorbed over the same titanium row. The molecules form molecular lines that run essentially perpendicular to the surface rows of bridging oxygen atoms, but they exhibit a wavy character that is similar to the case of pentacene described above [35]. Previous experiments performed by Reiss *et al.* using NEXAFS spectroscopy measurements revealed that the plane of the anthracene molecule in the monolayer is tilted by approximately 28° with respect to the surface; for naphthalene molecules, the angle reaches approximately 24° [38]. The acene molecules only weakly interact with the surface, and the TPD spectra collected for benzene, naphthalene and anthracene indicated that the adsorption energy increases almost linearly as the number of carbon atoms increases. These findings indicate that these molecules are all adsorbed in similar geometries [38]. Potapenko *et al.* also performed experiments with functionalized derivatives of anthracene molecules, in which one hydrogen atom was substituted by a chlorine atom [39]. The 2-chloroanthracene molecules were also observed to primarily adsorb between rows of bridging oxygen atoms and were rotated around molecular longer axis. The molecules weakly interact with the substrate, and cryogenic temperatures that cause immobilization of the molecules are required for imaging sub-monolayer coverages. Potapenko *et al.* also performed experiments that led to the tip-induced dissociation of 2-chloroanthracene molecules, in which the tunneling electrons that were injected into the σ states were driving dissociation reactions, contrary to gas phase dissociation, which may also occur when π states are excited. Therefore, electrons with energies greater than 2.5 eV are required for reactions on the surfaces to occur.

In addition to acene molecules, perylene and its derivatives have also been widely investigated on different surfaces. The adsorption of perylene on the (110) surface has been investigated by Simonsen *et al.* [40] with XPS (X-ray photoelectron spectroscopy), UPS (ultraviolet photoelectron spectroscopy) and NEXAFS spectroscopy techniques. The molecules were observed to lie more or less flat on the surface, and no supramolecular structures were observed. The molecules weakly interact with the substrate through vdW forces, which do not alter the electronic structure of the adsorbed molecules.

Schuster *et al.* have investigated the adsorption and self-assembly of other perylene derivatives, namely diidenoperylene (DIP) molecules, on the rutile (110) surface [41]. The authors observed that the molecules weakly interact with the substrate and that no chemical bonds are formed. The first layer does not exhibit any ordered structure. In thicker films, the molecules tend to nucleate in the form of molecular islands with the molecules oriented up-right.

**Figure 5:**
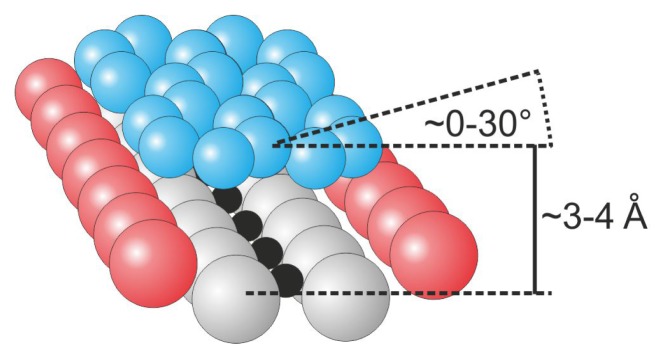
Hydrocarbon polycyclic molecule physisorbed on the TiO_2_(110) surface; the molecule is flat-lying with its plane slightly tilted with respect to the surface. The relatively high board altitude precludes strong binding and only allows for weak van der Waals and electrostatic attractions, color coding as in Figure 4, carbon atoms highlighted light blue.

In general, pure hydrocarbon polycyclic molecules only weakly interact with the titanium dioxide substrate, primarily through van der Waals and electrostatic forces. Consequently, the molecules are mobile on the surface and adsorb in a flat-lying geometry with the plane of the molecule slightly tilted from the plane of the surface at relatively high altitudes that reach 3–4 Å from the substrate surface, as schematically illustrated in Figure 5.

## 4. Polyaromatic Derivatives with Functionalized Groups

One of the most intensively studied functionalized perylene derivatives is the 3,4,9,10-perylene tetracarboxylic dianhydride (PTCDA) molecule. To date, this molecule has been examined on a broad variety of substrates that exhibit metallic, semiconducting and insulating properties (e.g., [42] and references therein). Tekiel *et al.* have investigated the adsorption of PTCDA molecules on the rutile (011) face [43].

**Figure 6:**
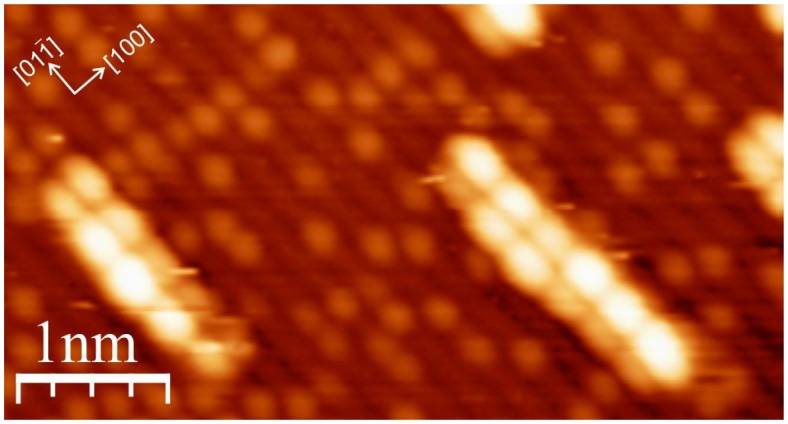
3,4,9,10-Perylene tetracarboxylic dianhydride (PTCDA) molecules forming quasi-one-dimensional molecular lines on the TiO_2_(011) surface: scanning tunneling microscopy (STM) image, bias voltage: +3.8 V, tunneling current: 1 pA; courtesy of Antoni Tekiel.

The authors observed that the molecules possessed very intriguing behavior and adsorbed in completely different configurations, depending on the deposition conditions. When the molecules were deposited on a substrate that was maintained at a temperature less than 350 K, the majority of the molecules could be observed as flat-lying with their longer molecular axis parallel to the surface rows and forming small clusters elongated parallel to the [01–1] direction. However, if the temperature of the substrate was maintained at a slightly greater temperature (approximately 350 K) during evaporation, almost all of the molecules were immobilized as single entities in a completely different geometry with the molecular axis perpendicular to the surface rows. A further small increase of the substrate temperature to 370 K during deposition resulted in the formation of well-ordered molecular lines that run along the surface rows with the molecules lying again in the initially described geometry (Figure 6). The mechanism for the surprising behavior is not known, but recent research performed by Cuan *et al.* [44] may shed some light on the possible processes. The results of the authors indicate that the interactions with the adsorbate induce restructuring of the (011) surface and leads to the formation of 1D molecular clusters.

Godlewski *et al.* also observed that the formation of 2D PTCDA ordered assemblies is highly limited and that some ordered 2D islands that exhibit limited lateral dimensions are only observed under specific conditions [45].

**Figure 7:**
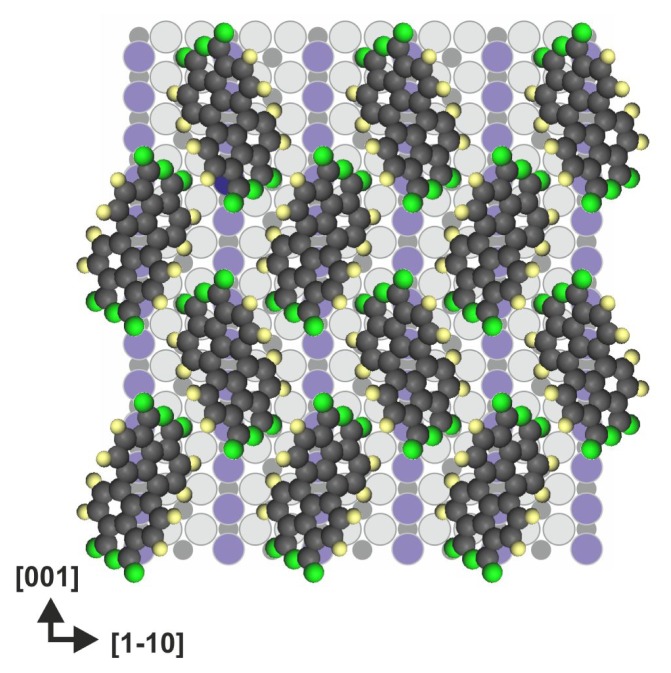
PTCDA molecules forming a herringbone-like structure on the TiO_2_(110) surface due to collectively acting dispersion forces [36], color coding: surface: violet–bridging oxygen, light gray–remaining oxygen atoms, black–titanium, atoms in the molecule: dark gray–carbon, green–oxygen, yellow–hydrogen.

Godlewski *et al.* investigated the adsorption of PTCDA molecules on the (110) surface and observed that the behavior of the molecules is completely different than that on the (011) face [36]. High-resolution STM studies supported by DFT (density functional theory) calculations, including semi-empirical dispersion corrections, revealed that at low coverage, the molecules are physisorbed on the surface in the flat-lying geometry centered over bridging oxygen atoms and with their molecular axis oriented along the surface reconstruction rows. However, increasing the coverage leads to the collective action of dispersion interactions, which surmount the chemisorption energy barrier. Consequently, a completely new, well-ordered herringbone like structure with strongly bent chemisorbed molecules is formed, which indicates that the intermolecular van der Waals forces could play a significant role in the self-assembly of molecular nanostructures (Figure 7). Recently, Ramalho and Illas also indicated that van der Waals forces may play a crucial role in the adsorption of organic molecules on rutile TiO_2_. The authors theoretically investigated the adsorption of azobenzene on rutile (110), and they reported that dispersion interactions significantly affect the binding energies of the isomers [46].

Cao *et al.* also investigated the PTCDA/TiO_2_(110) system, and they demonstrated that the LUMO is located directly above the bottom of the titanium dioxide conduction band, which means that the relative alignment is favorable for injecting electrons from photoexcited dye states into the sensitized substrate [47]. The authors also indicated that the growth mode of the molecules varies with increasing coverage.

Komolov *et al.* also investigated the adsorption of PTCDA on the TiO_2_(110) surface, and they indicated that a charge transfer occurs at the interface [48].

Schütte *et al.* investigated the adsorption of the PTCDI derivative, *i.e.*, *N*,*N*′-bis(1-hexylheptyl)-PTCDI, on the TiO_2_(110) surface [49]. Based on nc-AFM, STM and DFT modeling, the authors observed that the PTCDI derivatives adsorb over protruding bridging oxygen rows in a slightly tilted configuration with their longer molecular axis pointing along the surface rows. By varying the number of surface hydroxyls, the authors were able to conclude that the molecules are most likely pinned by surface defects. Moreover, the authors demonstrated that the molecules could be switched from the tilted geometry at one side of the bridging oxygen row to their mirror reflections in a controlled manner using a non-contact atomic force microscope at room temperature [50].

Simonsen has investigated the adsorption of 2,3,6,7,10,11-hexahydroxytriphenylene (HHTP) on the rutile TiO_2_(110) surface using XPS, UPS, NEXAFS spectroscopy and DFT modeling [51]. At a sub-monolayer coverage, the molecules were observed to lie in a flat geometry with the plane of the molecule tilted by approximately 32° with respect to the plane of the surface. However, when the monolayer is formed by the desorption of a multilayer film at elevated temperatures, the molecules within the first layer are oriented almost up-right at an angle of 80–85°. In this layer, the molecules are dissociated and bind to the surface titanium atoms. The layer does not exhibit any substantial ordering; however, the molecules in a submonolayer and in a monolayer are preferentially aligned along the [001] direction.

## 5. Phthalocyanines

Phthalocyanines are intensely colored macrocyclic organic compounds that are widely used in dyeing applications. The basic phthalocyanine molecule is a metal free compound, *i.e.*, H_2_Pc, that exhibits an intense blue-green color. Phthalocyanines form complexes with many elements from the periodic table, and the derivatives with metal atoms that substitute hydrogen in the central ring are widely used. Substituted Pcs with metal atoms are employed as organic dyes in dye-sensitized solar cells, in catalysis in the area of oxygen reduction reactions, as donors in optoelectronic devices, e.g., in field effect transistors [52], and in chemical sensing. In light of the above mentioned applications, it becomes clear that phthalocyanines are one of the most widely studied organic molecules on surfaces.

Phthalocyanines are structurally related to other macrocyclic pigments, *i.e.*, porphyrins which are also widely investigated on different single crystal surfaces. The studies on the interaction of phthalocyanines with titanium dioxide surfaces and the interface properties have primarily been driven by the idea of dye-sensitized solar cells, in which organic compounds play a sensitization role [53]. Therefore, phthalocyanines are the most widely investigated large polycyclic compounds on the titanium dioxide surfaces.

Palmgren *et al.* have studied the adsorption and interface properties of metal free phthalocyanines on the most well-known (110) surface using scanning tunneling microscopy and high resolution synchrotron based photoelectron spectroscopy [54]. They found that molecules lie flat and do not form any ordered structures even upon thermal treatment up to 500 K and adsorb as single entities with molecule arms pointing at the angle of 45° with respect to the [001] surface direction. The photoelectron spectra demonstrated that the molecules within the first layer bind strongly to the surface in a chemisorption fashion with the charge transfer from the molecule to the substrate. The major interaction takes place between delocalized electrons from π orbitals and the surface oxygen atoms and the authors find the situation unfavorable for sensitization of the oxide surface. The authors observed a striking difference in the interaction of the first layer and the second layer which exhibits bulk-like properties due to weak van der Waals bonding with the first molecular layer. The experiments demonstrated also the possibility to dehydrogenate molecules by thermal annealing at 500 K.

Apart from the metal-free phthalocyanines, also metal complexes have also been investigated on the surfaces of rutile. Palmgren *et al.* investigated the adsorption of iron phthalocyanines on both the (110) surface [55] and on top of a buffer layer formed by 4,4′-bipyridine molecules [56] using PES (photoelectron spectroscopy), XAS (X-ray absorption spectroscopy) and scanning tunneling microscopy. In the case of the FePc/TiO_2_(110) system, the behavior of the molecules is very similar to the previously described metal-free phthalocyanines. The interaction of the molecules with the substrate is strong, which leads to the chemisorption of nearly flat lying molecules that are oxidized and randomly distributed over the surface of the substrate. The strong interaction leads to a significant alteration of the electronic structure of the molecule and quenching of the HOMO-LUMO shakeup transitions in the carbon and nitrogen core level spectra. The second layer of molecules grows in an unordered island fashion, and spectroscopic data indicate that the original electronic properties of the FePCs are retained due to weak interactions with the underlying molecules. To avoid strong coupling with the substrate, which is very unfavorable for solar cell applications, the authors have proposed modifying the properties of the substrate with a buffer layer of bipy molecules [56]. The idea is related to the extensively growing field of functionalizing surfaces through the adsorption of different organic species that form well-ordered monolayers (e.g., see reference [22] and references therein). Palmgren *et al.* observed that 4,4′-bipyridine molecules form an ordered layer of almost up-right oriented molecules with one nitrogen atom pointing toward the vacuum interface. The adsorption of FePc molecules on top of the buffer layer results in decoupling of the molecules from the influence of the substrate, which was demonstrated by spectroscopic measurements. The formation of short lines running along the [001] direction (*i.e.*, the direction of the TiO_2_ reconstruction rows) composed from slightly tilted phthalocyanines that are presumably partially overlapping was observed. Yu *et al.* also investigated the adsorption of TiOPc on rutile (110) and on buffer layers formed by 2,2′-bipyridine, 4,4′-bipyridine, and 4-tert-butyl pyridine using X-ray absorption spectroscopy [57]. TiOPc molecules adsorbed directly on the rutile surface were observed to strongly bind with a significant charge transfer occurring into the substrate. This effect has been suppressed by the introduction of a buffer layer, which significantly reduced the charge transfer, but the efficiency of charge transfer reduction was altered with the use of different buffer molecules. This behavior is due to the formation of different dipoles on the molecule-substrate interface and alterations in the corresponding work functions.

General conclusions that could be drawn indicate that phthalocyanines strongly interact with the TiO_2_(110) surface and that their electronic structure is significantly affected, which is associated with a charge transfer from the molecule to the surface and the sensitization properties are quenched. Chemisorption in which surface oxygen atoms are involved leads to the immobilization of single molecules, which could be imaged using room temperature STM measurements. However, the second (and further) layer molecules weakly interact with the first layer and generally exhibit bulk-like properties. Similar behavior that resulted in the random adsorption of immobilized single molecular entities was observed by Godlewski *et al.* for phthalocyanines functionalized with copper atoms (CuPc molecules) on the (110) surface [58]. CuPc molecules were also investigated on other rutile faces. Wang *et al.* performed STM measurements of CuPc molecules on cross-linked TiO_2_(110)-(1 × 2) and TiO_2_(210) surfaces [59]. The experiments, which were primarily conducted at room temperature, demonstrated that the molecules also strongly interact with the substrate; however, the binding appears to be weaker than that for molecules on the (110) surface because some types of self-assembly processes were reported. On the cross-linked TiO_2_(110)-(1 × 2) surface, Wang *et al.* observed single molecules in flat-lying or tilted configurations, depending on the adsorption sites. The formation of some self-assembled molecular structures was reported when the coverage was increased and after thermal annealing. However, the overall degree of ordering was rather poor. At low coverages on the TiO_2_(210) surface, Wang *et al.* observed single molecules adsorbed on terraces, steps and on defects. Upon increasing coverage, the formation of molecular lines running along the [001] direction was reported. Neighboring molecular lines led to the creation of quasi-ordered 2D structures. Thermal annealing to 400 K resulted in the aggregation of 3D clusters.

**Figure 8:**
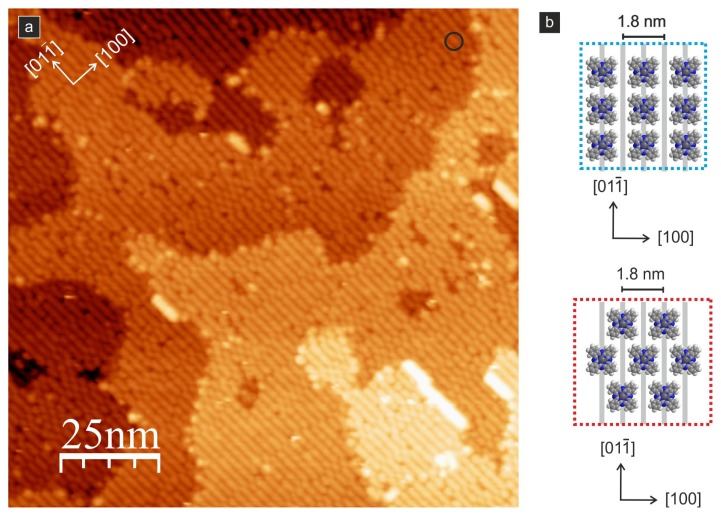
CuPc molecules forming a quasi-ordered phase on the TiO_2_(011)-(2 × 1) surface; (**a**) STM image, bias voltage: +3.0 V, tunneling current: 2 pA, black circle indicates single molecule within the layer; (**b**) structural models of two quasi-ordered phases, light gray lines indicate double zig-zag oxygen rows [45,60].

A further decrease of the molecule–substrate binding was observed for CuPc molecules on the (011) surface by Godlewski *et al.* [45,60]. The authors observed that single molecules are highly mobile on the surface, even when imaged at liquid nitrogen temperature. Moreover, the mobility of the molecules reflected the anisotropy of the surface structure, which resulted in the preferred direction of the molecule motion being parallel to the outermost double-oxygen zig–zag rows (*i.e.*, the [01–1] direction). This result is quite remarkable and indicates significant differences in the interactions with the substrate compared to other surfaces (e.g., the (110) surface), where all phthalocyanines are quite strongly immobilized. The authors observed that at low coverages, the molecules are almost free to move along the reconstruction rows at distances that are comparable to the separation of surface defects formed by hydrogen atoms. Therefore, the authors claim that these surface hydroxyls may form barriers for the motion of the molecules, at least when the system is cooled to cryogenic temperatures. Based on the high mobility and the possibility to easily desorb the molecules (at approximately 570 K, which is close to the sublimation temperature from CuPc powder), the authors concluded that the molecules are weakly bound to the surface. Consequently, the formation of large-scale ordered structures was observed for the first time. Due to intermolecular interactions when the monolayer coverage is reached, a quasi-ordered structure composed of flat-lying molecules is formed (see Figure 8).

Moreover, a further increase in the coverage results in the creation of the second layer of ordered islands that contains a mixture of flat-lying and up-right oriented molecules, which could be transformed into well-ordered structures of all flat-lying species upon thermal annealing at 420 K. A further increase of the annealing temperature resulted in a complete reorientation of the second layer into up-right standing molecules that exhibit bulk-like symmetry.

Ishida *et al.* investigated the adsorption of cobalt-substituted phthalocyanines (CoPc) on the rutile (110) surface and observed that the adsorption properties were strongly dependent on the deposition process [61]. The evaporation of molecules on a sample at room temperature results in mobile species, whereas post-deposition annealing to 400 K leads to the immobilization of molecules that are randomly distributed over the surface. Similar results were obtained during adsorption at elevated temperatures of up to 400 K, which suggests that the adsorption behavior changes significantly when thermal energy is provided and strengthens the molecule–substrate binding. STS measurements revealed that the molecules adsorbed on the surface introduce electronic states within the intrinsic band gap of the TiO_2_ substrate in the filled state region.

Ino *et al.* also demonstrated that charge transfer occurs for thin films of ZnPc molecules on the TiO_2_(110) surface [62].

## 6. Other Large Non-Planar Molecules

To date, a few different non-planar carbon or hydrocarbon polycyclic species have been investigated on rutile titanium dioxide surfaces. Godlewski *et al.* performed combined STM and nc-AFM studies of [11] anthrahelicenes on both (110) and (011) surfaces [63]. On the former surface, the molecules exhibit a large mobility that leads to clustering in quasi-hexagonal purely ordered islands that are attached to surface steps. In contrast, on the (011) face, the molecules are trapped on terraces and surface steps and no ordered structures are observed after increasing the coverage. Additionally, 2- and 3-dimensional clusters were observed.

Godlewski *et al.* investigated Violet Lander molecules that belong to the family of large non-planar species that are specially designed to act as molecular wires supported with spacer groups to increase the altitude of the polyaromatic board above the surface [45,64]. The authors investigated the adsorption on the (110) and (011) surfaces and observed that the molecules adsorb as single entities and adapt their conformation to the geometrical structure of the surface. Therefore, due to the large differences between both surfaces, a significant alteration of the adsorption geometry that strongly influences the mobility could be observed. The measurements revealed that the molecules may adapt one of two geometries on the (110) face, in which the board is either parallel or tilted from the direction of surface rows with molecular “legs” that are located in the trenches of the surface rows. Due to the walk-like manner of the molecule movement enabled by flexible σ bonds between the spacer groups and the molecular board, the different adsorption geometries result in a high mobility of molecules in a parallel geometry and highly reduced mobility of the tilted ones. Therefore, the key-and-lock effect, in which the mobility of the molecules could be switched by lateral manipulation between two geometries, is observed. On the (011) face, all of the molecules are observed in a tilted geometry that is characterized by a reduced mobility because of the different surface row spacings.

**Figure 9:**
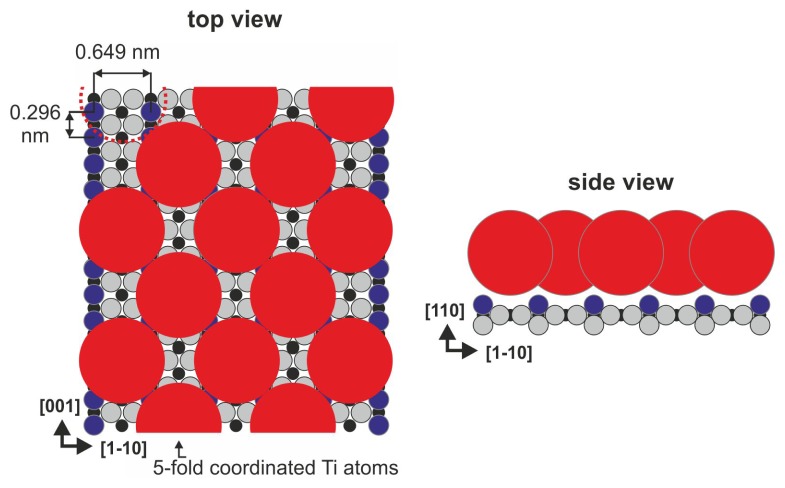
Structural model of an ordered layer formed by C60 molecules on the TiO_2_(110)-(1 × 1) surface [65].

Sanchez-Sanchez *et al.* investigated the electronic coupling between C60 molecules and the (110) surface of rutile titanium dioxide [65]. The molecules interact only weakly with the substrate by vdW binding, which is confirmed by XPS, NEXAFS spectroscopy and desorption from the surface at 600 K. Weak interaction with the substrate does not alter the electronic structure of the molecules. The C60 molecules, which are highly mobile on the surface, are confined by the anisotropic surface structure to form a close-packed p(5 × 2) superstructure (Figure 9). Within the layer, the molecules are located in two different adsorption sites, one of them being on top of 5-fold coordinated titanium atoms and the second in the bridging position between titanium atoms, and the molecules are vertically separated from the titanium atoms by approximately 3.2 Å. The molecules are spinning along the surface normal at room temperature, and molecular dynamics studies suggest that the high diffusion rate at the surface along the bridging oxygen rows is correlated with molecular rotation in such a way that the total angular momentum of two neighboring molecules is conserved.

Identical, well-ordered layers of C60 were also previously investigated using nc-AFM techniques by Loske *et al.* [66,67], who demonstrated the possibility of manipulating molecular islands with an AFM tip [68]. Loske *et al.* demonstrated that the molecules preferentially adsorb at surface steps at low coverages. Moreover, the molecular islands exhibit very nice ordering with a low density of defects, although the surface of the TiO_2_ substrate contains a considerable number of surface defects. This finding strongly suggests that the interaction of molecules with the surface and the surface defects is weak [66].

Fukui *et al.* investigated the adsorption of C60 molecules on a cross-linked TiO_2_(110)-(1 × 2) surface [69]. The molecules behave differently compared to the (110)-(1 × 1) substrate. During the initial stages of growth, single molecules are immobilized in the troughs between the outermost added rows, and the majority of them could be observed next to cross-links. The authors concluded that the immobilization occurs due to stronger interactions with the under-coordinated Ti atoms that are exposed at cross-links. Preferential adsorption of C60 to unsaturated Ti cations was also observed on the TiO_2_(100)-(1 × 3) surface [70]. With increasing coverage, the molecules tend to form domains that grow from the molecules anchored by cross-links and extend along the [001] direction, *i.e.*, the direction of added rows. When a monolayer is created, the molecules form rows. Overall, the interaction with the substrate is weak, but it is most likely stronger than in the case of the (110)-(1 × 1) surface. The authors concluded that by applying voltage pulses, chemical reactions occur between neighboring C60 molecules that form oligomers that run along the surface added rows.

**Figure 10:**
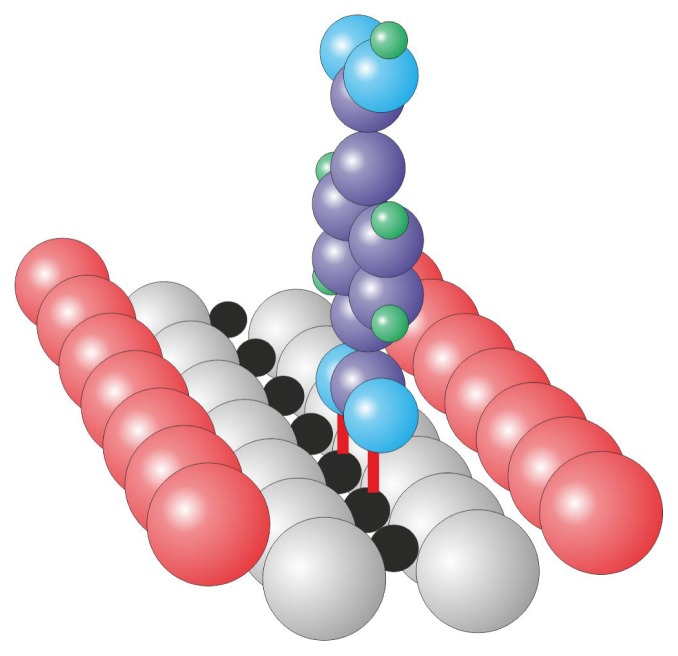
Bidentate adsorption behavior of molecules equipped with carboxyl groups; the presented up-right orientation is commonly observed within molecular layers. The red lines represent strong Ti–O chemical bonds, color coding: surface: red—bridging oxygen, light gray—remaining oxygen atoms, black—titanium, molecule: violet—carbon, light blue—oxygen, green—hydrogen.

Zinc-protoporphyrin adsorbed on the rutile TiO_2_(110) surface was studied using photoemission spectroscopy and near-edge absorption fine structure spectroscopy by Rienzo *et al.* [71]. The authors observed that the molecules initially lie flat but reorient into the up-right configuration when the coverage increases to a monolayer, which is similar to the case of TPA on TiO_2_ [22]. The molecules are strongly bound to the surface by a chemical bond between the oxygen atoms from the deprotonated carboxyl groups and the surface titanium atoms in a bidentate fashion, which is typical for molecules equipped with carboxyl groups (Figure 10 and [1,22,72–74] and references therein).

At low coverages, there is an interaction between the π electrons of the ring and the oxide surface, which allows for a flat lying geometry. However, when the coverage is increased, the strong chemical coupling between the deprotonated carboxyl groups dominates over the relatively weak ring-surface interactions, which results in the reorientation into the up-right position. Photoelectron spectra indicate that only the first layer of molecules becomes deprotonated upon interaction with the substrate and that the consecutive layers remain intact. The photoemission data indicate that the molecules chemically bound to the surface lose their central metal atoms, which leads to occupation of the center by two additional hydrogen atoms and has important consequences for the functionality of the system.

Weston *et al.* investigated large dipyrrin-based dye complexes on the rutile (110) surface [75]. The investigated molecules included bis(5-(4-carboxyphenyl)-4,6-dipyrrin)bis(dimethyl sulfoxide)ruthenium(II) (PY1) and bis(5-(4-carboxyphenyl)-4,6-dipyrrin)-(2,2′-bipyridine)ruthenium(II) (PY2). The PY1 and PY2 molecules were deposited using the electrospray deposition technique, and the system was characterized by photoemission spectroscopy. The molecules were observed to follow the adsorption path that is observed for smaller molecules that contain carboxylic groups. The molecules bind to the surface in a bidentate fashion in which both carboxylic groups are deprotonated and attached to 5-fold coordinated surface titanium atoms.

Senayake and Idriss indicated that irradiation with UV light could activate reactions that lead to the formation of high-molecular-weight organic compounds from formamide on single crystalline TiO_2_ [76].

## 7. Conclusions

In this review, we have summarized the major achievements from the last decade in the science of organic molecules self-assembled on single crystalline titanium dioxide. We have focused on large, polycyclic species and omitted the wide variety of smaller molecules that have also been investigated over the years. This omission is because there are several comprehensive papers devoted to smaller organic molecules; however, to the best of our knowledge, none of these papers refer to large species. We believe that it is advantageous to update the present achievements due to the growing interest in the applications for large molecules.

The experiments reveal that all large non-functionalized molecules that contain polyaromatic boards behave in a similar manner, in which they weakly interact with the surface primarily through van der Waals bonding, regardless of the molecule size, *i.e.*, the number of phenyl rings. The molecules tend to adsorb in a flat-lying geometry with a relatively high board altitude that reaches approximately 3–4 Å above the plane of the surface. The molecules exhibit great mobility and are stabilized on the surface at larger coverages by mutual interactions. Nevertheless, although the interaction with the surface is weak, the highly anisotropic surface structure results in a great anisotropy in the diffusion of the molecules. Substitution of the hydrocarbon components with functionalized units or metal atoms significantly changes the interaction with the surface. Functionalized molecules tend to form bonds with the titanium dioxide substrate; however, the formation of bonds strongly depends on the type of functional units and the plane of the surface. The latter observation arises from different exposures of surface titanium and oxygen atoms, which are involved in the molecule-substrate interaction. In principle, large molecules equipped with functional groups follow the adsorption path that has been previously described for small molecules and form similar bonds with the surface.

Large non-planar molecules, e.g., C60, are observed to be highly resistant to the influence of surface defects, which are inevitably present on the surface, thus allowing for the fabrication of well-ordered overlayers that exhibit large-scale lateral dimensions.

The considerable developments observed in scanning probe microscopy techniques over last 20 years provide precise tools for direct high-resolution imaging of a large variety of organic species on metal oxides. Moreover, new molecular deposition techniques, e.g., the electrospray deposition technique, enable research to be conducted on large molecules that cannot be thermally evaporated from molecular powder and contributes to the rapid development of research. Due to the remarkably wide area of applications, ranging from catalysis, electronic and optoelectronic devices, sensors and coatings to medical usage, the surface science of molecular nanostructures on titanium oxides appears to be one of the most important directions in the science of metal oxide surfaces.
